# Improvement of the Pour Plate Method by Separate Sterilization of Agar and Other Medium Components and Reduction of the Agar Concentration

**DOI:** 10.1128/spectrum.03161-22

**Published:** 2023-01-10

**Authors:** I. Terrones-Fernandez, P. Casino, A. López, S. Peiró, S. Ríos, A. Nardi-Ricart, E. García-Montoya, D. Asensio, A. M. Marqués, R. Castilla, P. J. Gamez-Montero, N. Piqué

**Affiliations:** a Department of Quality Control, Reactivos para Diagnóstico, S.L. (RPD), Barcelona, Catalonia, Spain; b Department of Genetics, Microbiology and Statistics, Biology Faculty, Universitat de Barcelona, Barcelona, Catalonia, Spain; c Department of Biochemistry and Biotechnology, Human Nutrition Unit, Universitat Rovira i Virgili, Reus, Catalonia, Spain; d Department of Pharmacy and Pharmaceutical Technology and Physical Chemistry, Faculty of Pharmacy and Food Sciences, Universitat de Barcelona, Catalonia, Spain; e Pharmacotherapy, Pharmacogenetics and Pharmaceutical Technology Research Group, Bellvitge Biomedical Research Institute (IDIBELL), L'Hospitalet de Llobregat, Catalonia, Spain; f Microbiology Section, Department of Biology, Healthcare and Environment, Faculty of Pharmacy and Food Sciences, Universitat de Barcelona (UB), Barcelona, Catalonia, Spain; g CATMech. Department of Fluid Mechanics, Universitat Politecnica de Catalunya, Terrassa, Catalonia, Spain; h Institut de Recerca en Nutrició i Seguretat Alimentària de la UB (INSA-UB), Universitat de Barcelona, Barcelona, Catalonia, Spain; University of Manitoba

**Keywords:** pour plate method, TSA, SDA, VRBG, recovery, selectivity, agar, separation of components, pour plate

## Abstract

Although the pour plate method is widely employed in microbiological quality control, it has certain drawbacks, including having to melt the culture medium before seeding. In this study, the preparation of the culture medium was modified by using a lower concentration of agar (10 g/L), which was separated from the nutrients during sterilization. The new protocol was assessed in media frequently used in microbiological quality control of food, cosmetics, and pharmaceutical products, with tryptic soy agar (TSA), Sabouraud 4% dextrose agar (SDA), and violet red bile glucose agar (VRBG). In comparison with the conventionally produced media, the modifications significantly improved the growth of Saccharomyces cerevisiae in SDA, Staphylococcus aureus, Salmonella enterica subsp. *enterica* serovar Typhimurium, and Candida albicans in TSA and Escherichia coli ATCC 8739 and ATCC 25922 and *S.* Typhimurium in VRBG. The modified VRBG was also more selective for Pseudomonas aeruginosa. Regarding physicochemical properties, a significantly lower pH was observed in TSA and VRBG and lower strength values in TSA. Sterilizing agar separately from the other components of the medium and reducing the agar concentration to 10 g/L can improve microorganism growth and enhance the selectivity of differential media in the pour plate method. These modifications could facilitate the automation of this culture technique.

**IMPORTANCE** In the era of rapid microbiological methods, there is a need to improve long-established culture techniques. Drawbacks of the pour plate method include having to melt each medium separately before seeding. For this technique, we demonstrate that separating the agar from the other components of commonly used media during sterilization and reducing the agar concentration to 10 g/L can enhance microbial growth. The new protocol could have advantages in routine laboratory practice because less agar is required and the same molten agar suspension can be used to prepare different media. Moreover, these modifications could facilitate the automation of the pour plate method.

## INTRODUCTION

Despite the technological advances that have allowed the development of rapid analytical techniques such as PCR or enzyme-linked immunosorbent assay (ELISA), microbiological quality control is still based on culture methods and the isolation of microorganisms. This classical approach remains the reference standard in the microbiological analysis of food, cosmetics, and pharmaceutical products (1–5). As culture methods are time-consuming and involve large volumes of material, previous preparation of medium, and a high staff workload (1, 6, 7), there is a need to improve and simplify them. A particularly effective way of facilitating routine laboratory work is automation (1, 6, 7).

A key step in classical bacterial culture is seeding onto solid media for the aerobic plate count. There are three main methods, the spread plate, spiral plate, and pour plate (8), which differ in their methodologies and in the volumes seeded. The spread plate technique is time- and material-consuming and susceptible to human error, which can compromise the accuracy of the enumeration (9). The introduction of the automated spiral pour method represented an important milestone for the routine microbiology laboratory, allowing significant savings of time and materials. It is particularly advantageous for the quantitative estimation of viable microorganisms in food (10, 11) because the sample is distributed in different concentrations on the same plate, avoiding the need for a prior serial dilution step.

The pour plate method allows the plating of higher volumes of sample (1 mL) by dispensing a liquid inoculum onto an empty petri dish, which is then flooded with a molten medium (12, 13), such as tryptic soy agar (TSA) or Sabouraud 4% dextrose agar (SDA), or a selective medium, such as violet red bile glucose agar (VRBG) (8, 14). While the colonies in a spread plate assay are distributed across the agar surface, in the pour plate method they are either embedded within the agar layer or formed on the agar surface (15, 16). The pour plate method is commonly used for the microbiological control of food, cosmetics, and pharmaceutical products (5, 12, 17, 18) and particularly for the enumeration of microorganisms with low microbiological limits (for example, Staphylococcus aureus in food samples or mesophilic aerobic bacteria in drinking water according to ISO 6222:1999 [19]).

Although the pour plate method is widely employed, it has some drawbacks (20, 21). It is more time-consuming than the spread plate technique and needs a constant temperature bath to maintain the molten medium at 48°C. If the medium is too hot, microorganisms may be destroyed, but if too cold, the medium may become lumpy once solidified (13, 22). Additionally, the reading of results is not straightforward, as colonies embedded in the medium are less visible, particularly in media with high opacity, such as Baird-Parker agar, used for the detection of S. aureus (23). In this regard, the use of lower concentrations of agar could improve the count of embedded colonies.

Moreover, it would be advantageous to avoid contact between the gelling agent (agar) and the other medium components (24), as interactions can lead to color changes, precipitate formation, or loss of gel strength, even when the medium is ready to use after sterilization by autoclave. Separation of agar from the other ingredients during autoclaving has been shown to enhance the growth of certain microorganisms, probably because it avoids the formation of growth-inhibiting components (25–27), such as reactive oxygen species (ROS), which can compromise the culturability of sensitive species (28). Furthermore, changes in the oxygen composition during the stirring and sterilization of ingredients can also improve the productivity of certain culture media (29).

Attempts to improve the pour plate method could therefore include the use of lower concentrations of agar to enhance colony visualization and the separate application of molten agar, which could improve the growth of certain microorganisms and facilitate the design of automated devices. Ideally, there should be minimal staff intervention in sample processing to reduce repetitive stress injuries and human error.

As sophisticated automatic equipment is inaccessible for many laboratories, especially in developing countries, there is a need to promote interaction between microbiology and engineering to find cutting-edge alternatives. Laboratories require a flexible and modular platform that would allow all aspects of the plating protocol to be customized according to batch size and other individual requirements, without compromising the quality of the culture media.

In this context, the aim of the present study was to improve the effectiveness of the pour plate method by modifying the preparation and formulation of the culture media in a way that could also facilitate their automation. The tested modifications were the separation of agar from the other medium ingredients during sterilization and reducing the content of agar. The effects on productivity and selectivity were assessed in the media most frequently used for quality control of food, cosmetics, and pharmaceutical products.

## RESULTS

In preliminary studies, a series of assays were performed in order to compare agar concentrations in the standard medium (with all components together). The results obtained showed no statistically significant differences between 1.0% and 1.5% media.

### Effect of separating the nutrient phase and gelling phase on the productivity of SDA.

**FIG 1:**
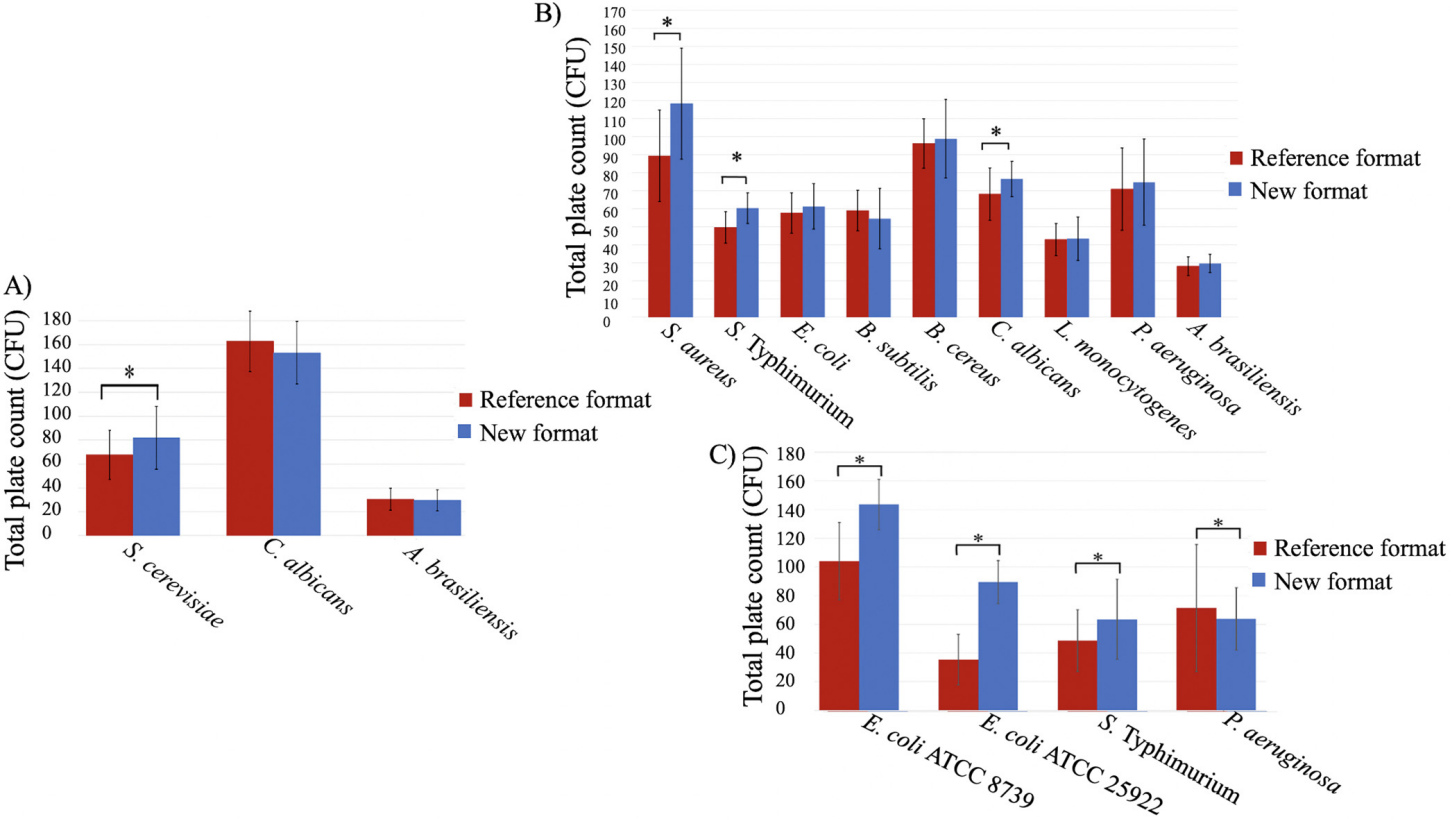
Bar graph showing mean CFU obtained in different culture media, conventional and modified, seeded by the pour plate method. (A) SDA for S. cerevisiae, C. albicans, and *A. brasiliensis*; (B) TSA for S. aureus*, S.* Typhimurium, E. coli, B. subtilis, B. cereus, C. albicans, L. monocytogenes, P. aeruginosa, and *A. brasiliensis*; (C) VRBG for E. coli ATCC 8739, E. coli ATCC 25922, *S.* Typhimurium, and P. aeruginosa.

**FIG 2:**
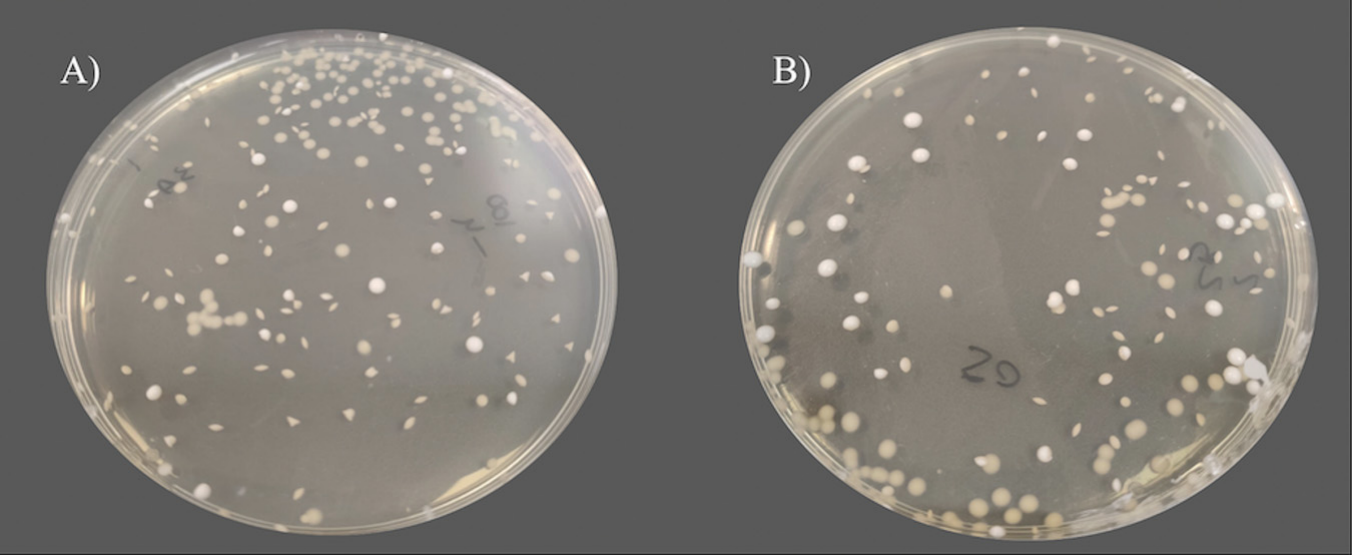
Comparison of growth in SDA when Saccharomyces cerevisiae was pour plated with (A) the reference SDA medium and (B) the modified SDA medium.

The modified SDA containing 10 g/L of agar and prepared with separately sterilized agar and nutrients was compared with the reference medium to test the influence of the modifications on productivity properties. Using the pour plate method, similar mean numbers of colony-forming unit (CFU) were obtained for Candida albicans ATCC 10231 (*P* = 0.145; analysis of variance [ANOVA]) and Aspergillus brasiliensis ATCC 16404 (*P* = 0.604; ANOVA) at day 3 in both types of media (Table 1 and Fig. 1A). In the case of Saccharomyces cerevisiae ATCC 9763, a statistically significant increase in the mean number of CFU at day 3 was reported in the modified medium (*P* = 0.001; ANOVA) (Table 1 and Fig. 1A). In terms of colony size, no significant differences were observed between formats for the three species in both surface and embedded colonies (Table 2 and Fig. 2). As expected, embedded colonies were smaller than colonies on the surface. For the fungus *A. brasiliensis*, only surface colonies were observed.

**TABLE 1:** Analysis of productivity and comparison of CFU counts of SDA media prepared conventionally and with modifications

Strain	Mean no. of colonies (CFU) on SDA medium^*a*^		*P* value^*b*^
	Reference	Modified	
S. cerevisiae ATCC 9763	67.54 ± 20.50	81.92 ± 26.26	0.001
C. albicans ATCC 10231	162.73 ± 25.40	153.13 ± 26.17	0.145
*A. brasiliensis* ATCC 16404	30.47 ± 9.21	29.73 ± 8.77	0.604

a Mean values ± standard deviations from triplicate samples are shown.

b ANOVA.

**TABLE 2:** Analysis of colony sizes (surface and embedded) in SDA media prepared conventionally and with modifications

Strain	Mean size (mm) values for SDA medium^*a*^				*P* value for^*b*^:	
	Reference medium		Modified medium			
	Surface colonies	Embedded colonies	Surface colonies	Embedded colonies	Surface colonies	Embedded colonies
S. cerevisiae ATCC 9763	2.33 ± 0.10	2.14 ± 0.80	2.68 ± 0.85	2.20 ± 0.59	0.421	0.851
C. albicans ATCC 10231	2.88 ± 0.48	2.98 ± 1.07	3.30 ± 0.92	2.55 ± 0.41	0.415	0.250
*A. brasiliensis* ATCC 16404	28.41 ± 4.79		26.52 ± 1.60		0.329	

a Mean values ± standard deviations of colonies measured are shown.

b Student’s *t* test for independent data.

### Effect of separating the nutrient phase and gelling phase on the productivity of TSA.

In TSA, a statistically significantly higher number of colonies was observed for S. aureus ATCC 6538 (*P* < 0.001; ANOVA), Salmonella enterica subsp. *enterica* serovar Typhimurium ATCC 14028 (*P* < 0.001; ANOVA), and C. albicans ATCC 10231 (*P* = 0.019; ANOVA) in the modified medium than in the reference medium (Table 3 and Fig. 1B).

**TABLE 3:** Analysis of productivity and comparison of CFU counts of TSA media prepared conventionally and with modifications

Strain	Mean no. of colonies (CFU) on TSA medium^*a*^		*P* value^*b*^
	Reference	Modified	
S. aureus ATCC 6538	89.47 ± 25.45	118.4 ± 30.82	<0.001
*S.* Typhimurium ATCC 14028	49.73 ± 8.74	60.47 ± 8.60	<0.001
E. coli ATCC 8739	57.77 ± 11.20	61.40 ± 12.57	0.086
B. subtilis ATCC 6633	59.10 ± 11.35	54.63 ± 16.81	0.104
B. cereus ATCC 11778	96.30 ± 13.74	98.97 ± 21.80	0.531
C. albicans ATCC 10231	68.20 ± 14.57	76.60 ± 9.88	0.019
L. monocytogenes ATCC 13932	43.03 ± 8.95	43.53 ± 12.04	0.805
P. aeruginosa ATCC 9027	70.93 ± 22.85	74.83 ± 23.91	0.386
*A. brasiliensis* ATCC 16404	28.28 ± 5.14	29.8 ± 5.08	0.717

a Mean values ± standard deviations from triplicate samples are shown.

b ANOVA.

As shown in Table 3, the mean numbers of CFU were similar in both media for Bacillus subtilis ATCC 6633 (*P* = 0.104; ANOVA), Escherichia coli ATCC 8739 (*P* = 0.086; ANOVA), Bacillus cereus ATCC 11778 (*P* = 0.531; ANOVA), Listeria monocytogenes ATCC 13932 (*P* = 0.805; ANOVA), Pseudomonas aeruginosa ATCC 9027 (*P* = 0.386; ANOVA), and *A. brasiliensis* (*P* = 0.717; ANOVA).

**FIG 3:**
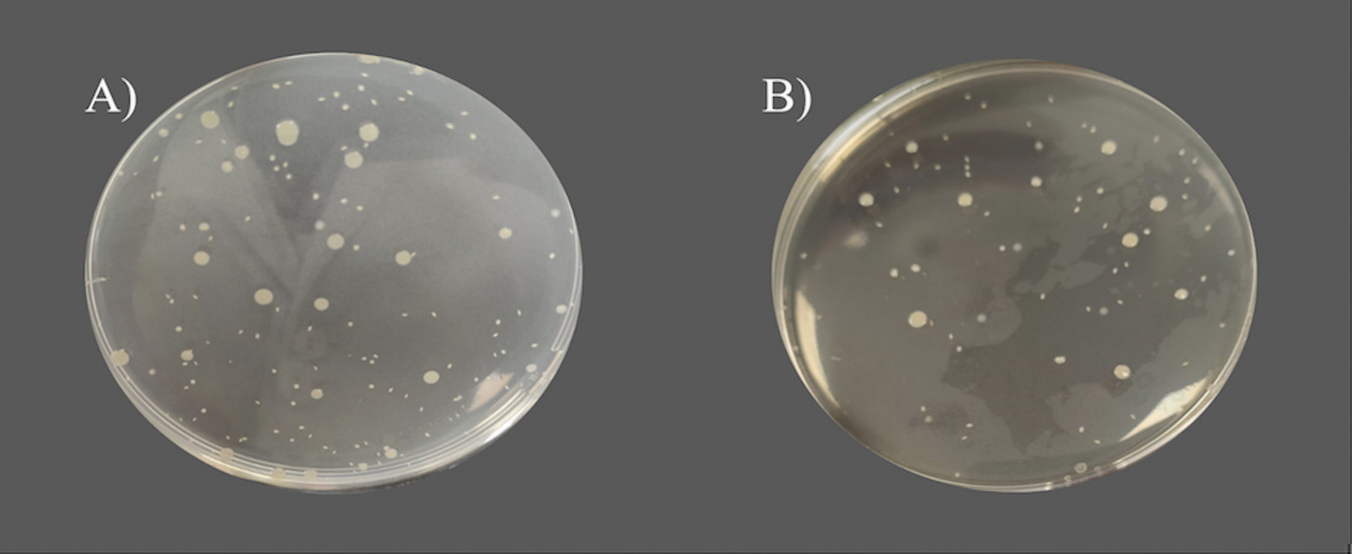
Comparison of growth in TSA when Staphylococcus aureus was pour plated with (A) the reference TSA medium and (B) the modified TSA medium.

In colony sizes, no statistically significant differences were observed between formats for all species studied, both in the surface colonies and in the embedded colonies (Table 4 and Fig. 3). As expected, embedded colonies are smaller than surface colonies.

**TABLE 4:** Analysis of colony sizes (surface and embedded) in TSA media prepared conventionally and with modifications

Strain	Mean size (mm) on TSA medium^*a*^				*P* value for^*b*^:	
	Reference		Modified			
	Surface colonies	Embedded colonies	Surface colonies	Embedded colonies	Surface colonies	Embedded colonies
S. aureus ATCC 6538	2.88 ± 0.48	1.48 ± 0.21	3.20 ± 0.26	1.39 ± 0.32	0.335	0.464
*S.* Typhimurium ATCC 14028	6.86 ± 0.27	1.48 ± 0.21	6.90 ± 1.40	1.33 ± 0.30	0.873	0.213
E. coli ATCC 8739	7.56 ± 1.40	1.26 ± 0.20	8.32 ± 1.31	1.30 ± 0.18	0.320	0.647
B. subtilis ATCC 6633	1.88 ± 0.42	0.91 ± 0.11	2.84 ± 1.48	1.03 ± 0.15	0.241	0.056
B. cereus ATCC 11778	5.20 ± 0.45	1.37 ± 0.41	4.80 ± 0.50	1.58 ± 0.44	0.122	0.284
C. albicans ATCC 10231	3.28 ± 0.23	3.45 ± 0.25	3.84 ± 0.70	3.34 ± 0.23	0.128	0.322
L. monocytogenes ATCC 13932	2.33 ± 1.04	2.14 ± 0.80	2.68 ± 0.85	2.20 ± 0.59	0.421	0.426
P. aeruginosa ATCC 9027	5.42 ± 1.01	1.32 ± 1.19	6.04 ± 0.91	0.88 ± 0.11	0.167	0.261
*A. brasiliensis* ATCC 16404	27.06 ± 1.92		30.35 ± 7.06		0.342	

a Mean values ± standard deviations of colonies measured are shown.

b Student’s *t* test for independent data.

For *A. brasiliensis,* only surface colonies were observed.

### Effect of separating the nutrient phase and gelling phase on the productivity of VRBG.

The productivity of the modified medium was statistically significantly higher than the reference medium for all the tested microorganisms, including E. coli ATCC 8739 (*P* < 0.001), E. coli ATCC 25922 (*P* < 0.001) and *S.* Typhimurium (*P* < 0.001), except for P. aeruginosa ATCC 9027, whose growth was statistically significantly lower (*P* = 0.034; ANOVA) (Table 5 and Fig. 1C). Regarding the selectivity (data not shown), complete inhibition was obtained in both media for S. aureus ATCC 6538 and Enterococcus faecalis ATCC 19433.

**TABLE 5:** Analysis of productivity and comparison of CFU counts of VRBG media prepared conventionally and with modifications

Strain	No. of colonies (CFU) on VRBG medium^*a*^		*P* value^*b*^
	Reference	Modified	
E. coli			
ATCC 8739	104.03 ± 26.95	143.50 ± 21.71	<0.001
ATCC 25922	35.30 ± 17.61	89.40 ± 14.95	<0.001
P. aeruginosa ATCC 9027	71.37 ± 44.31	63.73 ± 21.80	0.034
*S.* Typhimurium ATCC 14028	48.57 ± 21.53	63.43 ± 27.90	<0.001

a Mean values ± standard deviations of colonies measured are shown.

b Student’s *t* test for independent data.

**FIG 4:**
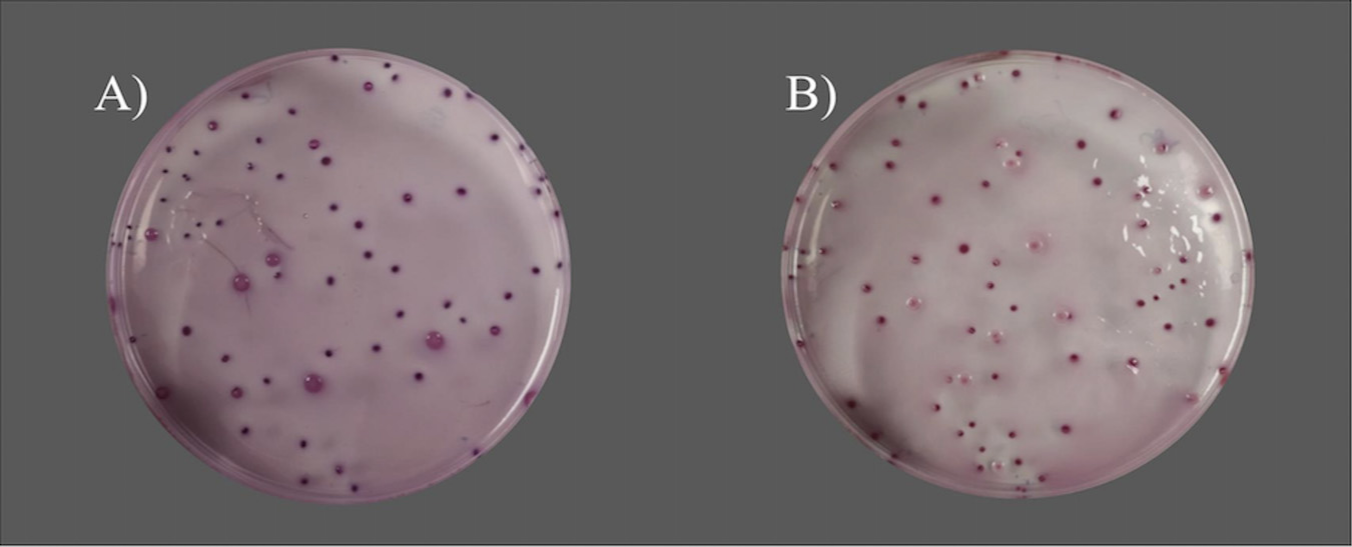
Comparison of growth in VRBG when Salmonella Typhimurium was pour plated with (A) the reference VRBG medium and (B) the modified VRBG medium.

The study of the colony sizes revealed no significant differences between formats for both surface and embedded colonies (Table 6 and Fig. 4). As expected, surface colonies were bigger than embedded colonies.

**TABLE 6:** Analysis of colony sizes (surface and embedded) in VRBG media prepared conventionally and with modifications

Strain	Mean colony size (mm) on VRBG medium^*a*^				*P* value for^*b*^:	
	Reference		Modified			
	Surface colonies	Embedded colonies	Surface colonies	Embedded colonies	Surface colonies	Embedded colonies
E. coli						
ATCC 8739	2.75 ± 0.92	1.11 ± 0.35	2.91 ± 0.38	0.88 ± 0.09	0.691	0.06
ATCC 25922	2.74 ± 0.24	1.31 ± 0.19	2.87 ± 0.21	1.18 ± 0.13	0.211	0.087
P. aeruginosa ATCC 9027	4.79 ± 1.50	1.21 ± 0.44	4.11 ± 0.96	0.97 ± 0.19	0.272	0.133
*S.* Typhimurium ATCC 14028	2.68 ± 0.18	1.47 ± 0.18	2.94 ± 0.34	1.60 ± 0.23	0.061	0.169

^a^Mean values ± standard deviations from colonies measured are shown.

^b^Student’s *t* test for independent data.

### Effect of separating the nutrient phase and gelling phase on medium pH.

**FIG 5:**
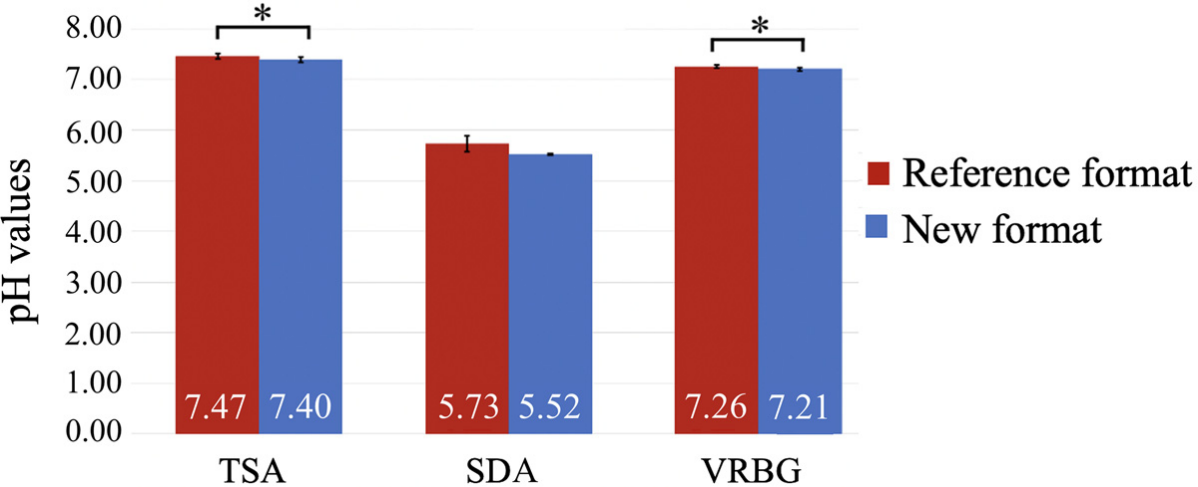
Mean pH values of the different culture media.

The medium pH was measured before sterilization and after autoclaving, when the culture medium was poured onto a sterile petri dish without a sample. After autoclaving, no statistically significant differences (*P* = 0.136; Student’s *t* test for independent data) were observed in the pH values of SDA according to the preparation (Fig. 5). In contrast, when the nutrient and gelling phases were separated, the pH was statistically significantly lower in TSA (*P* = 0.019; Student’s *t* test for independent data) and VRBG (*P* = 0.020, Student’s *t* test for independent data) (Fig. 5).

### Effect of separating the nutrient phase and gelling phase on the medium strength.

**FIG 6:**
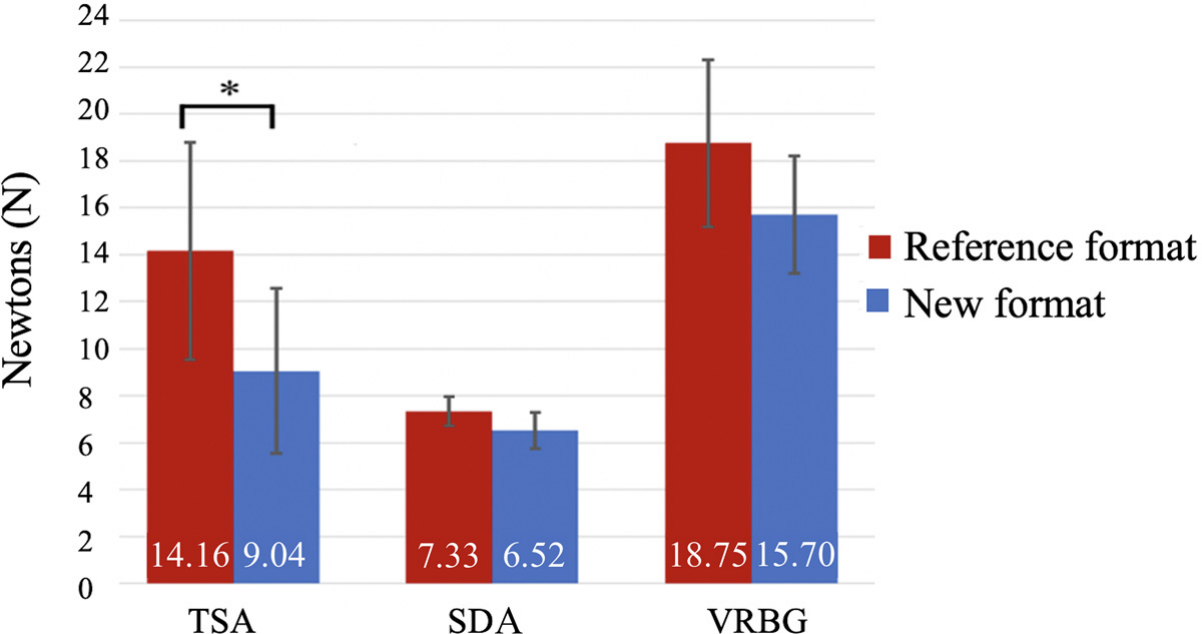
Mean strength values of the different culture media.

The strength values of TSA were significantly lower when the nutrient and gelling phases were sterilized separately and the agar concentration reduced (*P* = 0.008, Student’s *t* test for independent data) (Fig. 6). In contrast, the strength values of SDA (*P* = 0.115, Student’s *t* test for independent data) and VRBG (*P* = 0.077, Student’s *t* test for independent data) were not statistically significantly affected by the preparation or formulation.

## DISCUSSION

In the study of microorganisms and their viability, colony visualization on agar plates remains a fundamental technique (1–5). However, in the microbiological analysis of food, cosmetics, and pharmaceutical matrices, many factors can compromise the accuracy of the results (30), and CFU counts may be lower than the real number of viable bacterial cells. Moreover, the detection of viable but nonculturable (VBNC) bacteria on solid media is difficult (31–33), and several bacterial species cannot be cultured on agar plates. To overcome these limitations and facilitate the use of these culture techniques in routine laboratory practice, further research is necessary to improve seeding methods and enhance the productivity and selectivity of solid culture media (23, 26, 29, 34).

As a preliminary step in the separation of the gelling and nutrient phases, a series of assays were performed in order to compare agar concentrations in the standard medium (with all components together). The results obtained showed no statistically significant differences between 1.0% and 1.5% media. These results gave a way to decrease the agar concentration, as according to ISO 4833-1:2014 (13) a concentration from 9 g/L to 18 g/L of agar in the culture medium can be used.

In the present study, we sought to improve the productivity and selectivity of the assayed media in the pour plate method by the separation of agar from the other medium components during sterilization and reducing the concentration of agar. The use of less agar (10 g/L) improved the growth of embedded microorganisms regarding culture medium productivity; this may be due to increased availability of oxygen and nutrients when colonies are able to grow in an unconstrained formation because of the large pores in the matrix (35), although this was not assessed. Moreover, a lower concentration of agar facilitated the visualization of the embedded colonies. Moreover, as agar is extensively used in all solid media as a gelling agent, the use of smaller amounts would help to cut laboratory costs. The tested concentration (10 g/L) is accepted by ISO 4833, which states that agar levels can vary from 9 g/L to 18 g/L, depending on its gelling capacity (12).

Effects of separating medium components during sterilization have been reported previously (25–27). In a recent study, Kato et al. demonstrated that the separate sterilization of phosphate and agar improved the culturability of anaerobic microorganisms and increased the frequency with which phylogenetically novel microorganisms were isolated (27). This strategy also improved the culturability of reticent bacteria and slow-growing bacteria from natural environments (26). Chemical analysis of the culture media suggested that the formation of reactive oxygen species (ROS), particularly hydrogen peroxide, during agar autoclaving in the presence of other components, such as phosphate, was responsible for the lower growth of microorganisms on agar media (25–27).

The modifications in the present study (lower agar concentration and separation of components) were tested in three solid media commonly used in microbiological quality control: TSA for aerobic bacterial counts (36), SDA for the enumeration of yeast and molds (17), and VRBG for the detection and enumeration of bile-tolerant enterobacteria (18). The productivity, selectivity, and physicochemical properties of the modified media were compared with those of the conventionally prepared media (15 g/L of agar and sterilization of all components together) as a control.

The choice of VRBG as a selective medium is related to the importance given to the enumeration of *Enterobacteriaceae* in food (ISO 21528-2:2004 [18]). It is important that VRBG should be included in this investigation because it is only boiled when it is prepared because it can contain thermolabile compounds, so if only the gelling phase is melted and maintained in a water bath, degradation of thermolabile compounds could be avoided. These three media are widely used and manufactured in large amounts in comparison with more specific media.

Modified SDA yielded better growth for S. cerevisiae, while the growth of C. albicans and *A. brasiliensis* was similar to that of the reference medium. The improved growth of S. cerevisiae could be due to a lower degradation of glucose when the nutrients and gelling agent are autoclaved separately (37) because S. cerevisiae requires glucose to grow at higher rates (38). In the case of C. albicans, the ability of this yeast to grow on pour plate media with different agar concentrations has been previously described and reflects its capacity for invasive hyphal growth (39).

The modification of TSA, a general medium for the study of microorganisms, had a notable effect on S. aureus, *S.* Typhimurium, and C. albicans, resulting in significantly higher counts. This result could be attributed to a lower generation of ROS during separate autoclaving of components as described in previous studies (26, 27). The benefits of this procedure have been reported for conventional surface seeding, whereas the present study demonstrates its utility for the pour plate method.

The preparation of VRBG requires boiling for 1 min until all the components are well dissolved. With the new method, less time was needed to reach the boiling point (15 min versus 25 to 30 min), which may have reduced the glucose degradation associated with overheating of the media. This could explain the improvement in productivity observed in E. coli ATCC 8739, E. coli ATCC 25922, and *S.* Typhimurium when using the modified VRBG. A significantly higher productivity would improve the results in food quality control, where VRBG is commonly used as a selective medium for the enumeration of enterobacteria (18). The modifications did not alter selectivity against S. aureus and E. faecalis, but lower growth of P. aeruginosa was observed, probably due to an increase in certain selective conditions against this bacterium.

Regarding colony sizes in both surface and embedded colonies in all of the media studied, no significant differences were found as the moderate decrease in the agar concentration was proposed in order to maintain colony sizes in spite of other studies (35) where the changes in agar concentration affected this characteristic.

Key physicochemical properties, namely, pH and strength, were also compared in the modified and conventional media. During the preparation, the pH may need to be adjusted before autoclaving if the values stipulated by the ISO are not met. Compared to the control, pH values were statistically significantly lower in the modified TSA and VRBG and similar in SDA. In all culture media, the required values were maintained.

Using a durometer, the strength of the culture medium was assessed after it was cooled and poured onto a sterile petri dish without a sample. The limit for colony isolation without swarming of 1.0% (wt/vol) agar has been established by a previous study (40), and the better growth of microorganisms despite the significant lower strength values found only in TSA culture medium has been demonstrated. In TSA, the lower strength is correlated with a lower agar concentration. Although no significant differences in SDA and VRBG were observed, lower strength values were also found in these culture media. As mentioned, under these conditions, nutrients are more available for the embedded microorganisms, resulting in higher growth rates (35, 41). In future work, it would be of interest to study the effect of the oxygen percentages in conventional and modified agar matrixes (1.5% versus 1.0%) on the growth of different types of microorganisms. A reduced agar concentration has also been reported to favor motility and chemotaxis of mobile strains (35, 42). Although there are good prospects with these results, a further validation following ISO 16140-2:2016 (43) should be performed so this new format could be validated.

These favorable results suggest that a reduction in agar content could be a feasible way to lower laboratory costs in routine microbiological quality control. Our findings also indicate that a single suspension of molten agar can be used to prepare the different media, thereby reducing the material required and staff workload. Both of these modifications—component separation during autoclaving and the use of lower agar concentrations—may help lay the groundwork for the automation of the pour plate method.

A flexible modular system could be designed for the pour plate method, with automatic control of temperature and fluid dynamics properties for pouring, sample mixing, dilution, and sterile plate incubation management. Among the advantages of automation would be a reduction of repetitive stress injuries and involuntary errors, ensuring dosing without bubbles, homogeneity, and correct mixing of fluids of different density. In addition, less human intervention would help to provide an environment without cross contamination.

When talking about the usefulness of this type of test in developing equipment capable of automating the pour plate method, it refers to equipment where the gelling and nutrient phases are separated. In this way, the equipment would have 3 differentiated modules: (i) a module in which the agar (maintained in liquid state) and nutrient reservoirs are located and from which these liquids are pumped, (ii) a second module in which the mixture of both phases is homogenized, and finally (iii) a third module, which will be the one to dispense the culture medium on the plate.

In conclusion, these results demonstrate that improved microbial growth and selectivity can be obtained with the pour plate technique by reducing the agar concentration of the culture medium and separating the nutrients and gelling agent during sterilization. Another advantage is that the same molten agar suspension could be used for different media, further reducing the costs of materials and labor in routine laboratory practice.

## MATERIALS AND METHODS

### Manufacturing of culture media.

Tryptone soy agar (TSA), Sabouraud 4% dextrose agar (SDA), and violet red bile glucose agar (VRBG) were manufactured at Reactivos para Diagnóstico (RPD), S.L. (Sentmenat, Barcelona, Spain), from dehydrated ingredients using two methods and agar concentrations: (i) the reference media (all components together) were prepared conventionally using the standard composition with distilled H_2_O and the standard sterilization conditions following ISO 11133:2014 (44) (Table 7, Table 8, and Table 9), and (ii) in the modified culture media (nutrient and gelling phase separated), the nutrient phase (all components except agar) was separated from the gelling phase (agar) before autoclaving and maintained that way, and the amount of agar was reduced to 10 g/L (Table 7, Table 8, and Table 9).

**TABLE 7:** TSA medium composition

Component	Amt (g/L) in TSA medium	
	Reference	Modified
Casein peptone	15.0	15.0
Soy peptone	5.0	5.0
Sodium chloride	5.0	5.0
Agar	15.0	10.0

**TABLE 8:** SDA medium composition

Component	Amt (g/L) in SDA medium	
	Reference	Modified
d(+)-Glucose	40.0	40.0
Casein peptone	5.0	5.0
Meat peptone	5.0	5.0
Agar	15.0	10.0

**TABLE 9:** VRBG medium composition

Component	Amt (g/L) in VRBG medium	
	Reference	Modified
d(+)-Glucose	10.0	10.0
Gelatin peptone	7.0	7.0
Yeast extract	3.0	3.0
Bile salts	1.50	1.50
Sodium chloride	5.0	5.0
Neutral red	0.03	0.03
Crystal violet	0.002	0.002
Agar	15.0	10.0

The main differences between the two culture media were the separation of the components (nutrients and agar) and the agar concentration. For the reference media, TSA and SDA were autoclaved at 121°C for 15 min and VRBG was boiled for 1 min until completely dissolved. In the modified TSA and SDA, the nutrient phase and gelling phase were autoclaved separately at 121°C for 15 min (1 L of each phase concentrated 2-fold). In modified VRBG, the gelling phase (1 L of each phase concentrated 2-fold) was autoclaved at 121°C for 15 min and the nutrient phase was boiled for 1 min until completely dissolved (25 to 30 min to reach the boiling point in the reference medium and around 15 min in the new medium). Volumes of 200 mL of each sterilized phase were stored at room temperature. Molten agar was mixed with the nutrient phase when required and poured onto the petri dishes.

All culture media were, if necessary, pH adjusted (to pH values of 5.6 ± 0.2 for SDA, 7.3 ± 0.2 for TSA, and 7.4 ± 0.2 for VRBG) to achieve the physicochemical properties required for microbial growth. In the reference medium, the pH was adjusted for all components mixed together, whereas in the modified medium, it was only adjusted in the nutrient phase because agar by itself has a pH value of 6.8.

### Microorganism strains used.

The microbial strains used in the productivity and selectivity assays were obtained from a reference strain collection (American Type Culture Collection [ATCC]). The reference strains were subcultured once to obtain stock strains from which stock and working cultures were prepared. The strains used (Table 10) were those recommended by ISO 11133:2014/Amd 1:2018 for the quality control of TSA, SDA, and VRBG (44).

**TABLE 10:** Microorganisms and culture media tested

Culture medium	Assay type	Microorganism	Strain no.^*a*^	
			ATCC	WDCM
TSA	Productivity	Escherichia coli	8739	00012
		Candida albicans	10231	00054
		Pseudomonas aeruginosa	9027	00026
		Staphylococcus aureus	6538	00032
		Aspergillus brasiliensis	16404	00053
		Bacillus subtilis	6633	00003
		Listeria monocytogenes	13932	00021
		Salmonella enterica subsp. *enterica* serovar Typhimurium	14028	00031
		Bacillus cereus	11778	00001
SDA	Productivity	Aspergillus brasiliensis	16404	00053
		Candida albicans	10231	00054
		Saccharomyces cerevisiae	9763	00058
VRBG	Productivity	Escherichia coli	8739	00012
		Salmonella enterica subsp. *enterica* serovar Typhimurium	14028	00031
		Pseudomonas aeruginosa	9027	00026
		Escherichia coli	25922	00013
	Selectivity	Enterococcus faecalis	19433	00009
		Staphylococcus aureus	6538	00032

a ATCC, American Type Culture Collection; WDCM, World Data Center for Microorganisms.

### Inoculum preparation.

The stock culture strains were maintained in either TSA (bacteria) or SDA (molds and yeasts) slant tubes (RPD S.L.) under refrigeration. Working culture inocula were prepared according to ISO 11133:2014/Amd 1:2018 (44). Each strain was verified in a laminar flow cabinet before use in nonselective media (TSA for bacteria and SDA for molds and yeasts) and then in the appropriate selective and differential media according to ISO 17025:2017 (45), which were diluted in maximum recovery diluent (RPD S.L.) using turbidimetry (around 0.5 McFarland unit) (densitometer DEN-1B; Grant Instruments, Cambridgeshire, United Kingdom); serial dilutions were prepared using the same diluent. For the productivity assays, serial dilutions were prepared to seed 50 to 120 CFU per plate. For the selectivity assays (S. aureus and E. faecalis species), serial dilutions were prepared to seed ≥10^4^ CFU per plate according to ISO 11133:2014/Amd 1:2018 (44).

### Seeding method.

Samples of 1 mL were plated in triplicate onto sterile 90-mm petri dishes (polystyrene plastic) by the pour plate method in a laminar flow and biological safety II cabinet. Liquid culture medium was dispensed in 20-mL volumes at a temperature of 40 to 45°C in the petri dishes previously inoculated with the sample. In the productivity assays, plating was also performed by the spiral plate method to estimate the CFU in each dilution as a quality control (results not shown). For the pour plate method, the reference culture media were melted in a boiling water bath and then cooled to 40 to 45°C before being poured onto the plates. In the modified pour plate method, the nutrient phase was maintained at room temperature, while the gelling phase was maintained in a molten state in a water bath. The molten agar (50 to 55°C) was added directly to the nutrient phase solution, which on reaching the correct temperature (40 to 45°C) was poured onto the plates.

TSA plates were incubated in ovens (Binder GmBH) at 32.5 ± 2.5°C for 24 to 48 h (*European Pharmacopeia*, 10th ed. [5], and ISO 21149:2017 [36]), SDA plates at 20 ± 2.5°C to 25 ± 2.5°C for 2 to 5 days (*European Pharmacopoeia*, 10th ed. [5], and ISO 16212:2017 [17]), and VRBG plates at 37 ± 2.5°C for 24 to 48 h (ISO 21528-2:2004 [18]). For each medium and strain, seeding was repeated on 10 different days.

### Colony count.

The colony count was performed either manually or using an automatic colony counter (SphereFlash; IULmicro, Barcelona, Spain). The size of colonies (surface and embedded colonies counted separately) was obtained first in pixels and then converted to millimeters using the automatic colony counter and also the mobile application Pixel Measure 1.0 (Leroy Hopson Apps, Vietnam).

### Productivity assays.

Productivity assays were performed by the pour plate method according to the corresponding ISO (13, 17, 18, 36) and *European Pharmacopoeia*, 10th ed. (5), guidelines with the culture media required for each strain (Table 10). A microbial suspension containing 50 to 120 CFU (1 mL) was plated on sterile petri dishes, and then the culture medium was poured onto the petri dish and mixed with the sample.

### Selectivity assays.

Selectivity assays in VRBG were performed by the pour plate technique according to ISO 21528-2:2004 (18) and ISO 11133:2014/Amd 1:2018 (44) with S. aureus (ATCC, equivalent to WDCM 00032) and E. faecalis (ATCC 19433, equivalent to WDCM 00009), seeded at ≥10^4^ CFU (1 mL).

### Physicochemical properties.

To check that each culture medium had the required pH values, pH was measured with a pHmeter (Crison GLP21) before sterilization and after sterilization when the medium was poured onto a sterile petri dish. Additionally, the strength of the modified and reference media was measured (in newtons) with a durometer (DR) (Schleuniger Model 6D). Measurements were performed in triplicate on 10 different days.

### Statistical analysis.

All data were analyzed by the general linear model using SPSS v.28.0 (IBM Corp., Chicago, IL, USA). The mean and standard deviation were calculated for all measurements (number of colonies per plate). The numbers of CFU obtained (adjusted to a McFarland scale) in media prepared by both methods were subjected to analysis of variance (ANOVA) using the general linear model procedure to eliminate interday variability, due to the existence of two factors (plating day and medium preparation method). Adjustment to a McFarland scale ensures the Gaussian distribution of bacterial concentrations and allows a known concentration in each dilution.

pH and strength values in different media, both reference and modified, were compared using the Student's *t* test for independent data. In all tests, the significance (α) level was set as 0.05 and means and standard deviation were calculated for all means (number of colonies/plate).
